# Human mesenchymal stem cells in the tumour microenvironment promote ovarian cancer progression: the role of platelet-activating factor

**DOI:** 10.1186/s12885-018-4918-0

**Published:** 2018-10-19

**Authors:** Tong Gao, Yi Yu, Qing Cong, Yisheng Wang, Mingming Sun, Liangqing Yao, Congjian Xu, Wei Jiang

**Affiliations:** 1 Department of Gynecology, Obstetrics and Gynecology Hospital, Fudan University, 419 Fangxie Road, Shanghai, 200011 People’s Republic of China; 2 Shanghai Key Laboratory of Female Reproductive Endocrine Related Diseases, 413 Zhaozhou Road, Shanghai, 200011 People’s Republic of China

**Keywords:** Ovarian cancer, Mesenchymal stem cells (MSCs), Platelet-activating factor (PAF), Microenvironment

## Abstract

**Background:**

The tumour microenvironment conferred by mesenchymal stem cells (MSCs) plays a key role in tumour development and progression. We previously determined that platelet-activating factor receptor (PAFR) was overexpressed in ovarian cancer cells (OCCs) and that PAF can promote ovarian cancer progression via PAF/PAFR-mediated inflammatory signalling pathways. Evidence suggests that MSCs can secrete high concentrations of PAF. Here, we investigated the role of PAF/PAFR signalling in the microenvironment mediated by MSCs and OCCs and its effect on cancer progression.

**Methods:**

The PAF concentrations in the culture media of MSCs, OCCs and co-cultured MSCs and OCCs were determined by ELISA. The effects of MSCs on OCCs in vitro were assessed on cells treated with conditioned medium (CM). The expression and phosphorylation of key proteins in the PAF/PAFR signalling pathway were evaluated. In vivo, MSCs/RFP and SKOV3 cells were co-administered at different proportions to nude mice by interscapular injection. Mice in the WEB2086 group were intraperitoneally injected with the PAFR antagonist WEB2086 at a dose of 1 mg/kg^.^d for the duration of the animal experiments. Tumour progression was observed, and the weight and survival time of mice were measured. The PAF concentration in peripheral and tumour site blood was determined by ELISA.

**Results:**

High concentrations of PAF were detected in CM from MSCs and MSCs co-cultured with OCCs. Both types of medium promoted non-mucinous OCC proliferation and migration but had no effect on mucinous-type OCCs. These effects could be blocked by PAFR inhibitors. The expression and phosphorylation of key proteins in the PAF/PAFR pathway significantly increased upon treatment with PAF and MSC-CM. In vivo, the tumour volume was larger following co-injection of SKOV3 cells and MSCs/RFP than following injection of SKOV3 cells alone. The tumour-promoting effect of MSCs/RFP was blocked by the PAFR antagonist WEB2086. Serum PAF concentrations significantly increased in co-injected mice.

**Conclusion:**

Our results suggest that the tumour-promoting effect of MSCs on OCCs via their cross-talk in the tumour microenvironment was, at least in part, mediated by the PAF/PAFR pathway, suggesting a new target for the treatment of ovarian cancer.

## Background

In women, ovarian cancer (OC) is the seventh most commonly diagnosed cancer worldwide [1]. Although OC accounts for only 3% of all cancer diagnoses, 6% of cancer-related deaths are caused by OC, making it the fifth leading cause of cancer-related mortality in women [2]. Given the lack of screening for detection of early-stage OC, an estimated 85% of patients with OC present with advanced-stage (III/IV) disease [3]. The current treatment entails surgery followed by chemotherapy with a combination of taxanes and platinum [4]. Unfortunately, approximately 70% of women with advanced OC relapse within a few years after treatment and die due to the development of drug resistance [5]. Therefore, a thorough understanding of the development and progression of OC and more effective strategies for the treatment of OC are urgently needed.

The tumour microenvironment, which is the combination of noncancerous cells and molecules produced by all cells present in the tumour, plays an important role in tumour behaviour, including proliferation, invasion or metastasis and response to therapy [6]. Adult human mesenchymal stem cells (hMSCs) home to tumour sites and participate in the formation of the tumour microenvironment. MSCs can migrate to injured tissue and play an important role in inflammation and trauma [7].

One of the hallmarks of cancer is the effect of inflammation on the tumour microenvironment [8]. In OC, an inflammatory state is considered a risk factor and can be associated with OC development, drug resistance, and metastasis [9]. Platelet-activating factor (PAF) is an important pro-inflammatory activator of platelets, neutrophils, macrophages, lymphocytes, and endothelial cells [10], which are often essential microenvironmental components interacting with cancer cells. The role of PAF and its receptor (platelet-activating factor receptor, PAFR) in tumours has been investigated in recent years. PAF and PAFR are involved in oncogenic transformation, anti-apoptosis, metastasis and angiogenesis in several types of cancers [11]. We demonstrated that PAF/PAFR signalling is commonly activated in non-mucinous ovarian cancer cells (OCCs) and contributes to cancer progression and drug resistance. PAF can activate PAFR and activate multiple downstream signalling pathways, specifically through steroid receptor coactivator/focal adhesion kinase (Src/FAK) and downstream targets in cancer cell proliferation and invasion [12–14].

hMSCs in bone marrow can secrete high concentrations of PAF (50 times higher than those in serum) in some physiological or pathological environments [15]. Current reports show that MSCs can play different roles in different tumours or even in the same tumour [16]. We postulate that hMSCs (or cancer-associated fibroblasts derived from hMSCs) in the OC microenvironment could promote cancer progression via the PAF/PAFR pathway.

In this study, we measured the concentration of PAF in the culture media of hMSCs and hMSCs co-cultured with OCCs. We examined the effect of MSC-CM on OCCs and the involvement of the PAF/PAFR pathway both in vitro and in vivo. Our results show that cross-talk between cancer cells and MSCs in the OC microenvironment was partly mediated by the PAF/PAFR pathway.

## Methods

### Cell culture and chemical reagents

The OCC lines OVCA433, RMUG-L, 3AO, OMC685, TOV112D, and DOV13 were kindly provided by Bin Ye (Department of Obstetrics and Gynecology and Reproductive Biology, Laboratory of Gynecologic Oncology and Epidemiology, Brigham and Women’s Hospital, Harvard Medical School, Boston, Massachusetts, USA). These cell lines, along with SKOV3 and ES-2 cells (purchased from the Cell Bank of the Chinese Academy of Science, Shanghai, China), were cultured at 37 °C in a humidified 5% CO_2_ atmosphere in Roswell Park Memorial Institute-1640 (RPMI-1640) medium with 10% foetal calf serum (Gibco, Invitrogen, Carlsbad, CA) (serum-free medium was used when measuring the concentration of PAF in the medium), 100 IU/ml penicillin G, and 100 mg/ml streptomycin sulfate (Sigma-Aldrich, St. Louis, MO). MSCs (purchased from Cyagen Biosciences, Guangzhou, China) were cultured at 37 °C in a humidified 5% CO_2_ atmosphere in adult bone marrow mesenchymal stem cell (MSC) complete medium. PAF, ginkgolide B (GB), and WEB2086 (PAFR antagonist) were purchased from Sigma-Aldrich. Human and mouse PAF enzyme-linked immunosorbent assay (ELISA) kits were purchased from Groundwork Biotechnology Diagnosticate (San Diego, CA). The stem cell induction differentiation kit was purchased from Cyagen Biosciences (Guangzhou, China).

### PAF assay

SKOV3, 3AO, ES2, CAOV3, OMC685, and RMUG-L cells, representing different pathological types of OC, were cultured alone or co-cultured with MSCs. Conditioned medium (CM) was collected at 12 h, 24 h, 48 h, 3 d and 7 d and centrifuged at 10,000 rpm for 10 min. For a better comparison, we also collected peritoneal fluids and serum from 8 patients with endometriosis and 6 patients with high-grade ovarian serous carcinoma. These samples were collected from the hospital tissue bank for the purpose of research. This study did not involve the diagnosis and treatment of patients. Informed consent was waived by the ethics committee of Obstetrics and Gynecology Hospital of Fudan University. Tail vein blood and tumour site blood were collected from nu/nu mice in each group, and serum PAF concentrations were measured. All samples were analysed separately using a specific enzyme immunoassay kit (Groundwork Biotechnology Diagnosticate, San Diego, CA) to determine the concentration of PAF. The assay was performed according to the manufacturer’s instructions. The PAF concentration was evaluated in duplicate using a PAF standard curve. The sensitivity of the assay allowed the detection of up to 15 pg/ml. When necessary, the samples were diluted in the assay buffer. All data were presented as the mean ± standard deviation.

### OCC proliferation in MSC-CM

MSCs were cultured in 6-well plates at 1*10^6^ cells per well. The culture medium was collected after 24 and 48 h and filtered through a 0.22-μm filter to remove cell debris. This medium was used as CM for OCC culture. OCCs were treated with different concentrations of PAF (0, 1, 10, and 100 nmol/l). After 6 h, cell proliferation was detected by a 3-(4,5-dimethylthiazol-2-yl)-2,5-diphenyl tetrazolium bromide (MTT) assay. Cell lines representing different pathological types of OC were cultured in CM. After 24 h and 48 h, 10 μl MTT dye solution was added to each well, and the plates were incubated at 37 °C for 4 h in a humidified chamber. After incubation, 100 μl solubilisation stop solution was added to each well. One hour after the addition of the solubilisation solution, the contents of the wells were mixed, and the absorbance value at 570 nm was measured.

### Transwell migration assay

Transwell assays were performed to measure cell migration abilities. A total of 1*10^4^ SKOV3 cells were seeded into 24-well Transwell plates in serum-free medium. Culture medium supplemented with 10% FBS was added to the lower chamber. Cells were incubated for an additional 48 h. At the end of the experiments, non-migrating cells on the upper surface were scraped off using a cotton swab. The cells on the lower surface were fixed with 4% paraformaldehyde for 10 min and then stained with Crystal violet solution at room temperature for 15 min. The cells were imaged and counted in five randomly selected microscopic fields (20×).

### Immunofluorescence

SKOV3 cells cultured with or without MSC-CM were fixed with 100% methanol for 6 min at − 20 °C and then washed with phosphate-buffered saline (PBS) and stored at − 4 °C until use. The cells were permeabilised by incubation in PBS containing 0.3% Triton X-100 and 5% goat serum for 30 min. A polyclonal antibody against FAK was used at a 1:100 dilution, and a FITC-conjugated goat anti-rabbit secondary antibody (Invitrogen) was used at a 1:200 dilution. The primary antibody was incubated overnight at − 4 °C, and the secondary antibody was incubated for 2 h at room temperature. Images were captured with an Olympus DP 71 camera (Tokyo, Japan). The magnification level was 40×.

### Western blot analysis

Cellular extracts were prepared in modified radioimmunoprecipitation assay (RIPA) buffer (50 mM Tris–HCl pH 7.4, 1% NP-40, 0.25% Na-deoxycholate, 150 mM NaCl, 1 mM ethylenediaminetetraacetic acid (EDTA), 1 mM phenylmethylsulfonyl fluoride (PMSF), and protease inhibitor cocktail). The protein concentrations of cellular extracts were measured using a Bio-Rad protein assay kit. Then, cellular extracts were subjected to sodium dodecyl sulfate-polyacrylamide gel electrophoresis (SDS-PAGE). Proteins were transferred to polyvinylidene fluoride (PVDF) membranes. After blocking for 1 h at room temperature in 5% bovine serum albumin (BSA), blots were probed with the primary antibody at a 1:1000 dilution and incubated overnight at 4 °C. Subsequently, blots were washed three times and incubated for 1 h at room temperature with a 1:5000 dilution of peroxidase-conjugated secondary antibodies. Following three washes, immunoreactive bands were detected using electrochemiluminescence (ECL).

### Labelling of MSCs

MSCs were labelled with red fluorescence protein (RFP) by transfection with the vector pERFP-N1 (Clontech), and after 2 weeks of selection with G418 (400 μg/ml), stable MSCs/RFP were acquired and ready for in vivo experiments.

### Animals and treatment

Female BALB/c nude mice (4 to 6 weeks old) were obtained from the Laboratory Animal Center of the Shanghai Institutes for Biological Sciences of the Chinese Academy of Sciences and housed in a pathogen-free environment and used for OCC xenograft experiments. All animal studies were conducted strictly in accordance with protocols approved by the Ethics Committee for Animal Experimentation of Fudan University. Xenografts were established by subcutaneous injection of a total of 2*10^6^ cells in the shoulder of nude mice in the following experimental groups (LS: left shoulder, RS: right shoulder): 1. MSCs/RFP only were injected subcutaneously; 2. LS: MSCs/RFP only, RS: SKOV3 cells only; 3. LS: MSCs/RFP, RS: SKOV3 cells + MSCs/RFP, SKOV3 cells: MSCs/RFP = 1:1; 4. LS: SKOV3 cells, RS: SKOV3 cells; 5. LS: SKOV3 cells, RS: SKOV3 cells + MSCs/RFP, SKOV3 cells: MSCs/RFP = 1:2; 6. LS: SKOV3 cells + MSCs/RFP, RS: SKOV3 cells + MSCs/RFP, SKOV3 cells: MSCs/RFP = 1:2; 7. LS: SKOV3 cells + MSCs/RFP, RS: SKOV3 cells + MSCs/RFP, SKOV3 cells: MSCs/RFP = 1:1; and 8. LS: SKOV3 cells + MSCs/RFP, RS: SKOV3 cells + MSCs/RFP, SKOV3 cells: MSCs/RFP = 10:1. Animals were randomly divided into two groups. Mice in the WEB2086 group were intraperitoneally injected with the PAFR antagonist WEB2086 at a dose of 1 mg/kg^.^d for the duration of the animal experiments, while mice in the control group were injected with dimethyl sulfoxide (DMSO). Tumour sizes were measured 3 times per week. The tumours were measured using callipers, and tumour volumes were calculated using the following formula: tumour volume (mm^3^) = (tumour length) × (tumour width)2/2. The weights of mice were recorded 3 times per week.

### Preparation and analysis of tissues and histology

All mice were euthanised by CO_2_ inhalation followed by cervical dislocation. Subcutaneous tumour tissues were removed and embedded in a 1:4 dilution of optimum cutting temperature (OCT) compound in PBS. Fresh-frozen tumour sections (5-μm thick) were mounted on glass slides, and the distribution of MSCs/RFP in the tumour tissue was observed under a fluorescence microscope. For histological analysis, OCT-embedded tissue sections were sectioned for haematoxylin and eosin (HE) staining. Formalin-fixed paraffin-embedded tissue sections were sectioned for immunofluorescence analysis of PAFR. Histopathological analysis was performed by a pathologist with expertise in human and murine malignancies.

### Statistical analysis

All experiments were performed at least three times. The data are expressed as the “mean ± SD”. Wherever appropriate, the data were also subjected to unpaired two-tailed Student’s t-tests. Differences were considered significant when *P* < 0.05.

## Results

### High concentrations of PAF were detected in MSC-CM

**Fig. 1:**
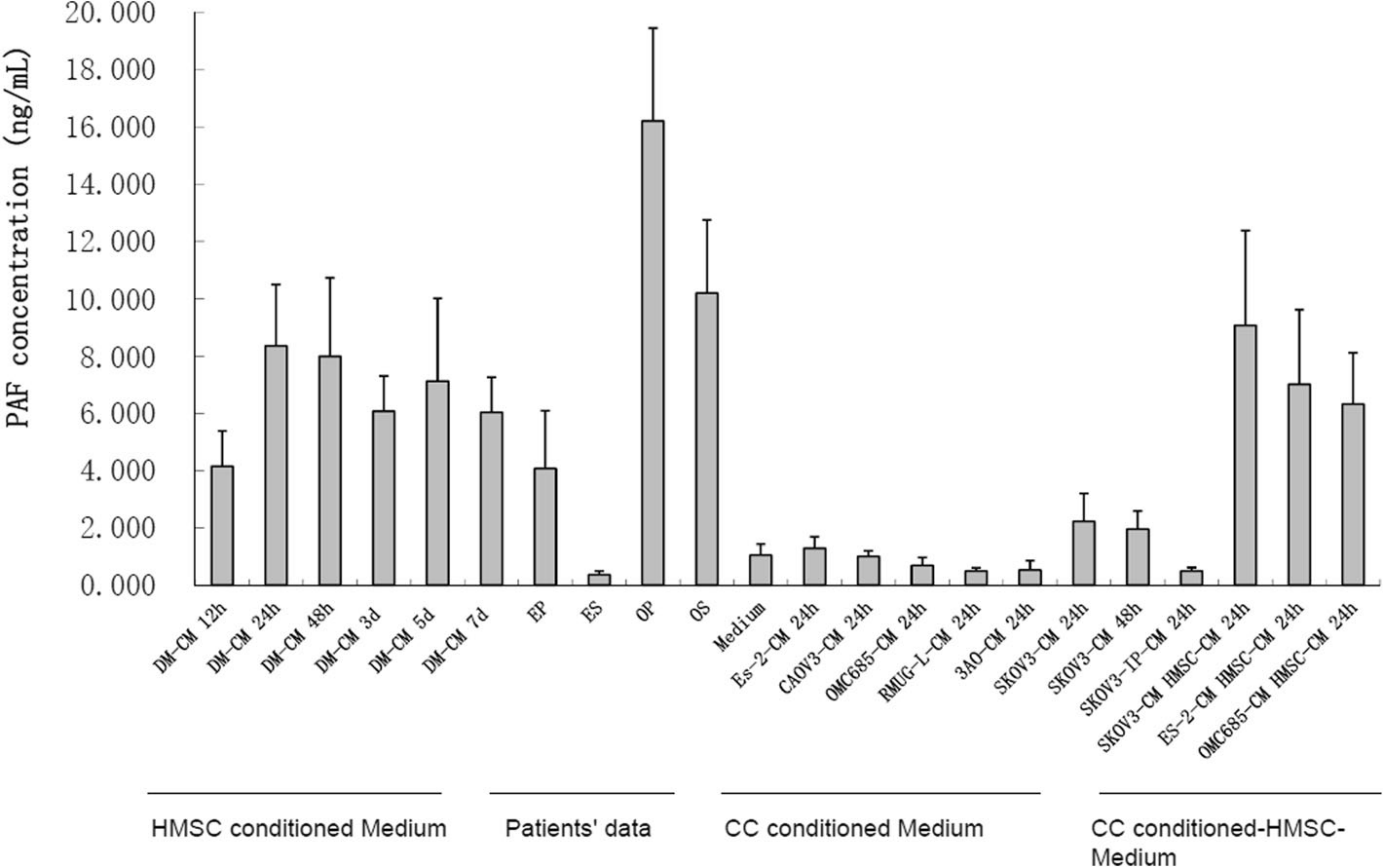
High concentrations of PAF were detected in MSC-CM. MSCs and OCCs secreted different concentrations of PAF. MSCs and different types of OCCs were cultured in serum-free medium. High concentrations of PAF were detected in MSC-CM and MSC-OCC-CM. Non-mucinous OCCs secreted a low concentration of PAF, while mucinous OCCs did not secrete PAF. The PAF concentration was measured in the peritoneal fluid and serum of patients with OC (6 patients) and endometriosis (8 patients) as a positive control. Bars represent the average of triplicates ± SD for media and Mean ± SD for patient samples. EP: peritoneal fluid of endometriosis patients. ES: serum of endometriosis patients. OP: peritoneal fluid of OC patients. OS: serum of OC patients

We first detected the PAF concentration in CM from MSCs, OCCs and MSCs co-cultured with OCCs using a PAF ELISA kit. As shown in Fig. 1, MSCs and OCCs secreted different concentrations of PAF. At the same time points, the concentration of PAF secreted by MSCs was higher than that secreted by OCCs, peaking at 24 h and then gradually decreasing. Non-mucinous OCCs such as ES2, CAOV3, and SKOV3 cells secreted low concentrations of PAF, while mucinous OCCs such as OMC685, 3AO, and RMUG-L cells did not secrete PAF. These results were consistent with those obtained in our previous study. High concentrations of PAF were detected in the ascites and serum of OC patients and in the peritoneal washing fluid of endometriosis patients, but very low concentrations were observed in serum samples from endometriosis patients. The consistently high PAF concentrations observed in MSC-CM and MSC-OCC-CM indicated that MSCs can secrete high concentrations of PAF when cultured alone or co-cultured with OCCs, consistent with the results of Denizot [15] et al.

### The effect of MSC-CM on OCC proliferation and the inhibitory effects of GB

**Fig. 2:**
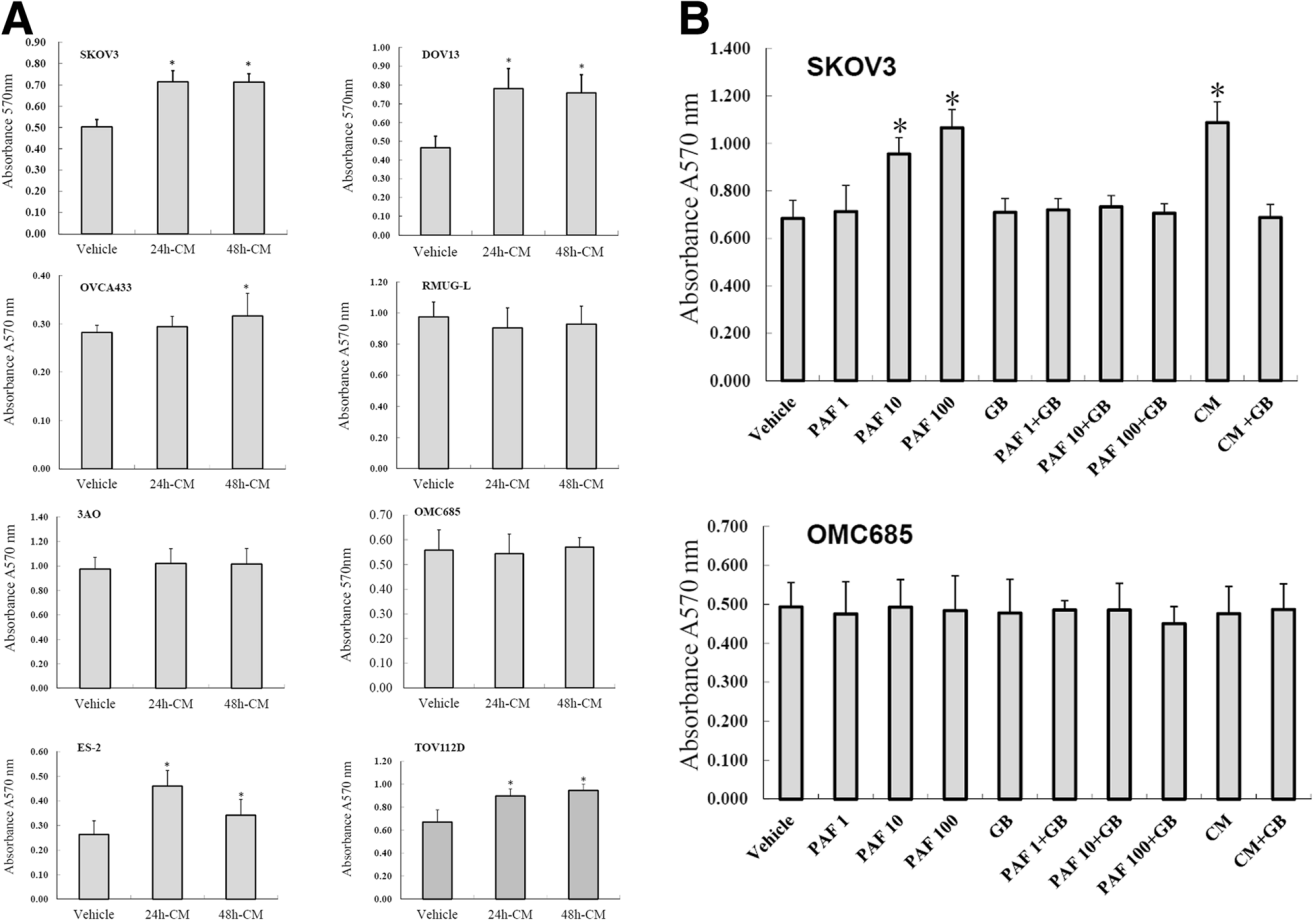
The effect of MSC-CM on OCC proliferation and the inhibitory effects of GB. MSC-CM promoted the proliferation of non-mucinous OCCs such as SKOV3, DOV13, OVCA433, ES2 and TOV112D cells but had no effect on mucinous OCCs such as RMUG-L, 3AO, and OMC685 cells. No significant differences were observed between 24 h and 48 h. **a** The non-mucinous OCC line SKOV3 showed dose-dependent responses to PAF treatment, a strong response to MSC-CM and significant increases in cell proliferation. The proliferation-inducing effect of MSC-CM was almost the same as that induced by stimulation with PAF at a high concentration (100 ng/ml). Compared with treatment with PAF alone and vehicle control, treatment with the PAFR antagonist ginkgolide B (GB) (100 μmol/l) significantly blocked PAF-induced and MSC-CM-induced cell proliferation. **b** The proliferation of the mucinous OCC line OMC685 (with negative PAFR expression) was not affected by PAF treatment and MSC-CM. “*” indicates a statistically significant difference between different groups

MSC-CM promoted the proliferation of PAFR-positive ovarian serous carcinoma cell lines such as SKOV3, DOV13, and OVCA433 and clear cell carcinoma cell lines such as ES2 and TOV112D. No significant differences were observed between 24 h and 48 h. However, MSC-CM had no significant effect on the proliferation of PAFR-negative mucinous OCC lines such as RMUG-L, 3AO, and OMC685 (Fig. 2a). To determine the role of PAF in MSC-CM-induced proliferation of non-mucinous OCCs, we performed further studies in the PAFR-positive cell line SKOV3 and the PAFR-negative mucinous cell line OMC685. SKOV3 and OMC685 cells were cultured in MSC-CM. Cells were treated with different concentrations of PAF (0, 1, 10, and 100 nmol/l) as the positive control, and the PAFR-specific antagonist GB was used at a concentration of 100 μmol/l to block the PAF/PAFR pathway. The proliferation of SKOV3 cells was significantly increased after 24 h of treatment with PAF (10 and 100 nmol/l) and MSC-CM, but the PAFR-negative mucinous OCC line OMC685 showed no response. The proliferation-inducing effect of MSC-CM was similar to the effect of 100 nmol/l PAF. In addition, compared with PAF treatment alone, 100 μmol/l GB administered with PAF almost completely inhibited PAF-induced cell proliferation in these cancer cells (Fig. 2b). These data indicated that MSC-CM promotes the proliferation of non-mucinous OCCs at least in part via the PAF/PAFR pathway.

### The effect of MSC-CM on SKOV3 migration and the inhibitory effects of GB

**Fig. 3:**
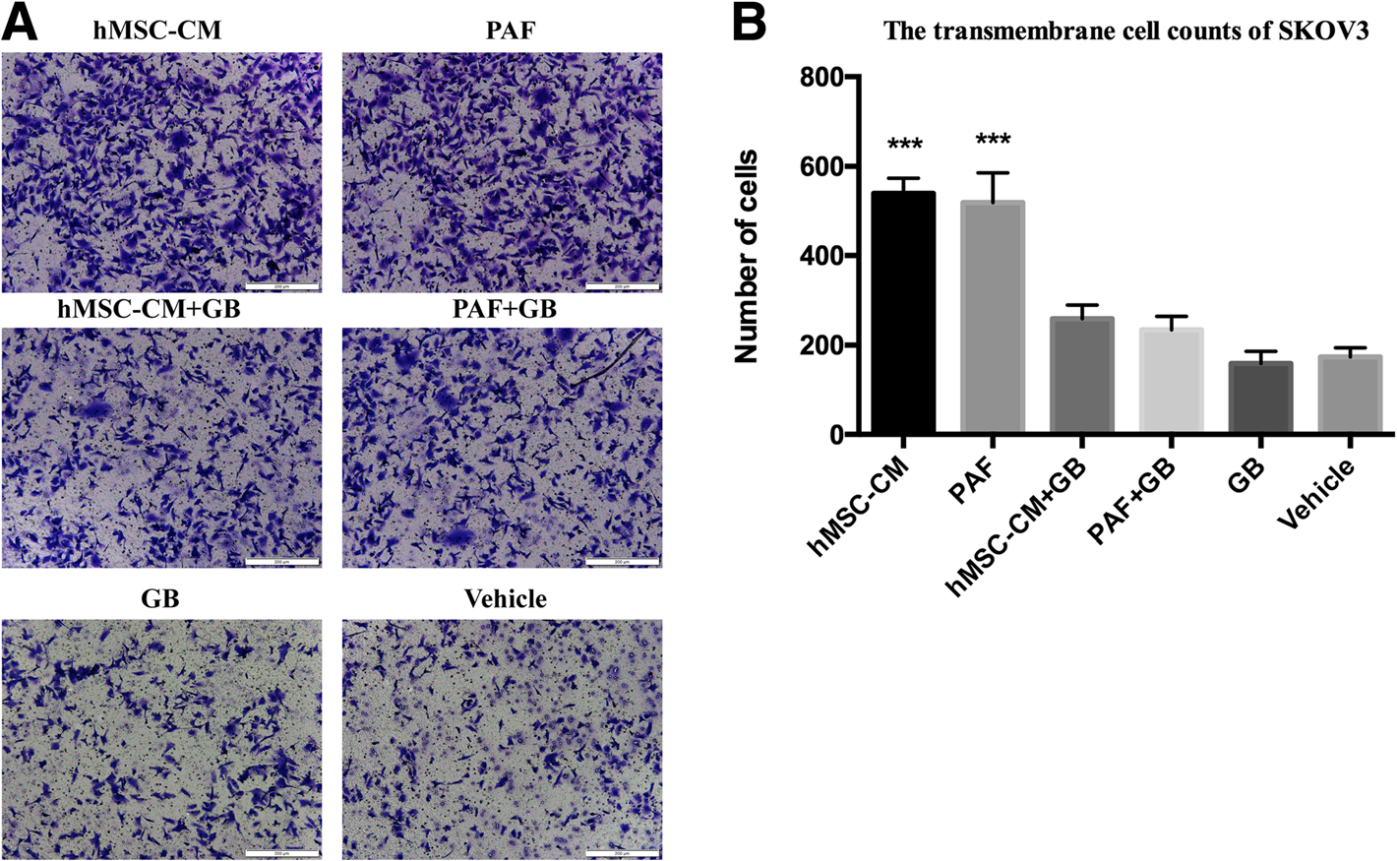
The effect of MSC-CM on OCC migration and the inhibitory effects of GB. MSC-CM promoted the migration ability of SKOV3, and the migration-inducing effect of MSC-CM was similar to the effect of 100 nmol/l of PAF. Compared with PAF treatment alone, 100 μmol/l GB almost completely inhibited PAF-induced cell migration in cancer cells (**a** and **b**)

SKOV3 cells were treated with MSC-CM and 100 nmol/l PAF, and Transwell assays were performed. MSC-CM promoted the migration ability of SKOV3, and the migration-inducing effect of MSC-CM was similar to the effect of 100 nmol/l PAF. Compared with PAF treatment alone, 100 μmol/l GB administered with PAF almost completely inhibited PAF-induced cell migration in cancer cells (Fig. 3).

### The effect of MSC-CM on key proteins of the PAF/PAFR pathway in OCCs

**Fig. 4:**
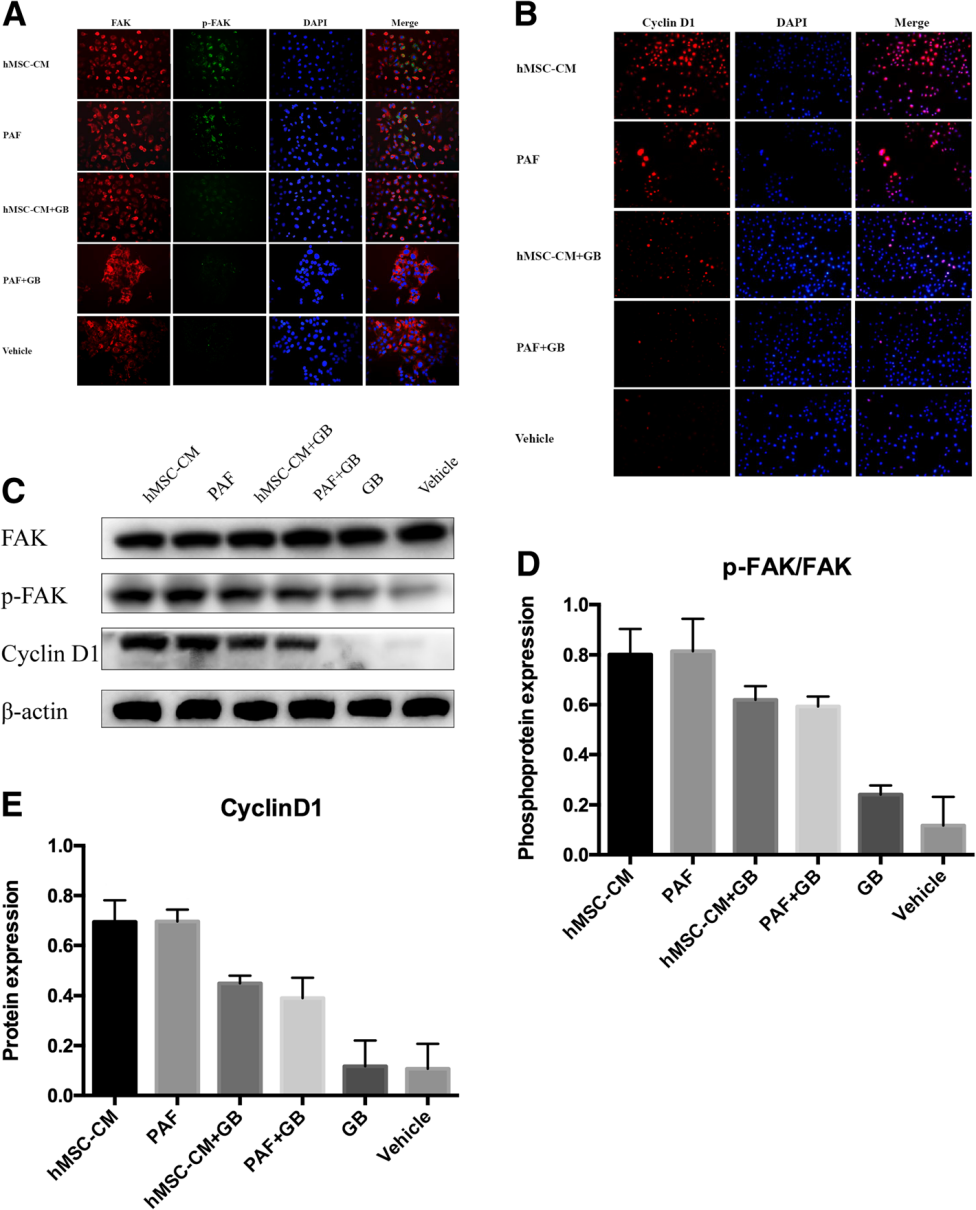
The effect of MSC-CM on key proteins in the PAF/PAFR pathway in OCCs. As described in our previous study, key proteins involved in the PAF/PAFR pathway, cyclin D1 and phosphorylated FAK, were induced by MSC-CM. For the immunofluorescence staining of phosphorylated FAK (**a**) and cyclin D1 (**b**) in SKOV3 cells, after 24 h of incubation with MSC-CM or PAF with or without GB, the cells were labelled with polyclonal antibodies against cyclin D1 and phosphorylated FAK overnight and then incubated with a fluorescent secondary antibody for 1 h and stained with DAPI for 10 min (with magnification 40×). MSC-CM induced cyclin D1 expression and FAK phosphorylation, and these effects were blocked by GB. These effects were confirmed by Western Blot analysis (**c**, **d** and **e**)

To further investigate the possible molecular mechanisms of the regulatory functions of MSC-CM in OC, we examined the expression of cyclin D1 and phosphorylated FAK, which are key proteins in the PAF/PAFR pathway as described in our previous study [12], using specific antibodies. SKOV3 cells were cultured in MSC-CM and treated with PAF (100 nmol/l) alone or with the PAFR antagonist GB (10 μmol/l). As shown in Fig. 4a and b, the expression of cyclin D1 and the site-specific phosphorylation of FAK simultaneously increased in cells cultured in MSC-CM and treated with 100 nmol/l PAF compared with that in control cells, and the addition of GB reduced the staining intensity of both proteins. As shown in Fig. 4c, d and e, the Western blot analysis was consistent with the immunofluorescence analysis. These data indicated that MSC-CM activated key proteins involved in PAF/PAFR signalling in SKOV3 cells, further demonstrating that the effect of MSC-CM is PAFR-dependent.

### The effect of MSCs on OC via the PAF/PAFR pathway in vivo

**Fig. 5:**
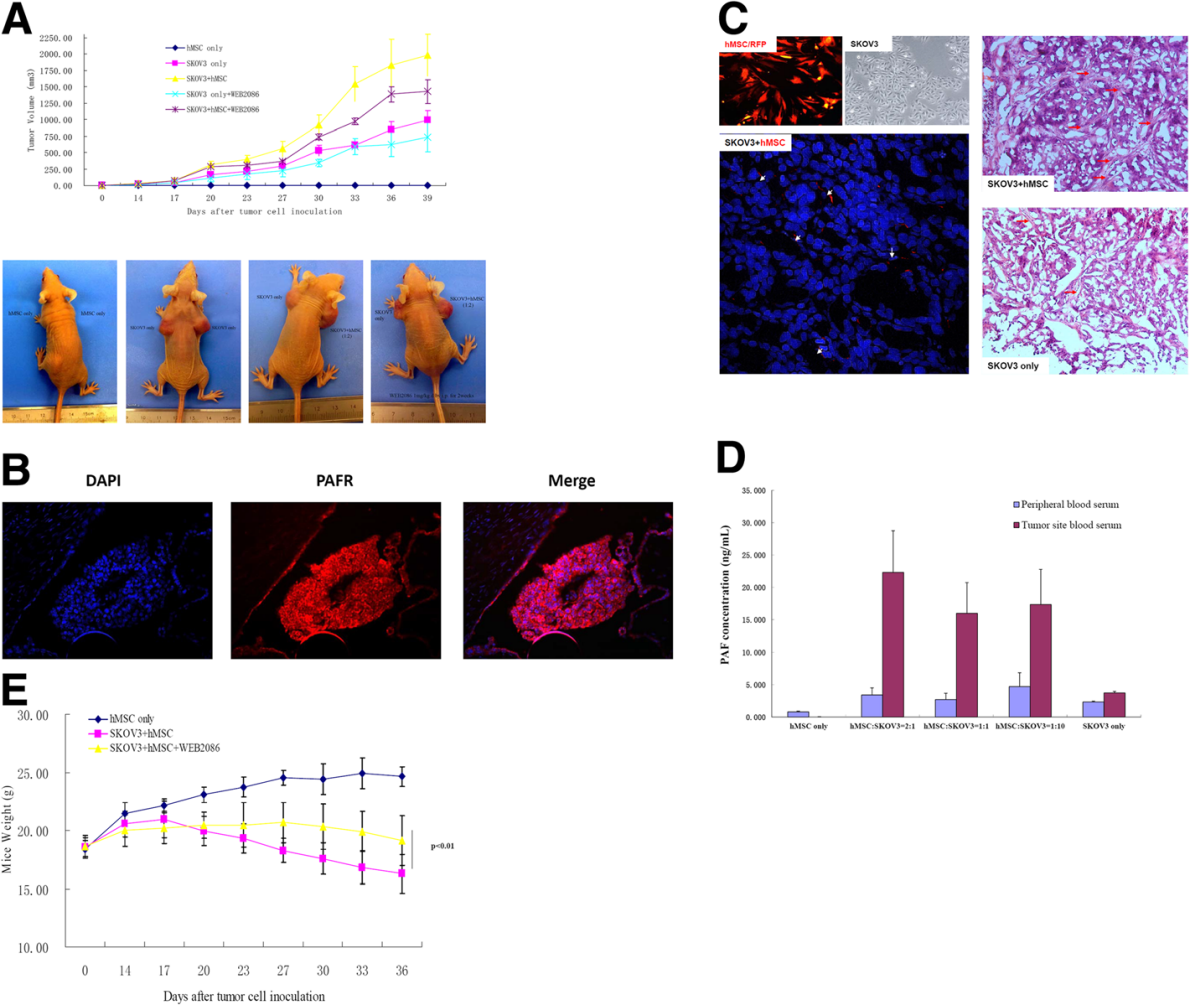
The tumour-promoting effect of MSCs on OC via the PAF/PAFR pathway in vivo. **a** The tumour volume was determined in mice, and the data represent the average (+SD). Student’s t-test was used to compare tumour sizes among the different groups; *p* < 0.05 indicates a statistically significant difference. MSCs alone were not tumourigenic, while they significantly promoted the growth of SKOV3-derived subcutaneous tumours. The PAFR antagonist WEB2086 blocked this effect. Compared with the SKOV3 + WEB2086 group, the SKOV3 + MSC + WEB2086 group exhibited a significantly larger tumour volume, indicating that the PAFR inhibitor could not completely inhibit the tumour-promoting effect of MSCs. The following row contains representative photographs of mice injected with (from left to right) 1. LS: MSCs/RFP, RS: MSCs/RFP; 2. LS: SKOV3 cells, RS: SKOV3 cells; 3. LS: SKOV3 cells, RS: SKOV3 cells + MSCs/RFP (1:2); and 4. LS: SKOV3 cells, RS: SKOV3 + MSCs/RFP (1:2), treated with WEB2086 at 1 mg/kg.d by intraperitoneal injection for 2 weeks. **b** High PAFR expression in tumour tissue from mice injected with SKOV3 cells verified by IFC (20×). **c** Frozen sections were stained with DAPI and observed under a confocal microscope. MSCs/RFP could be visualised in the tumour stroma in the co-injection groups. HE-stained frozen tumour sections were photographed under a microscope (20× or 40×). **d** After MSC injection, the concentration of PAF in the tumour site was significantly higher than that in peripheral blood; a 10-fold upregulation of PAF was observed when MSCs and SKOV3 cells were co-injected at a ratio of 2:1 compared with that when SKOV3 cells were injected alone. **e** The weight of mice was recorded 3 times per week. Mice in the WEB2086 group had higher weights than did mice in the DMSO group (SKOV3 + MSC)

Finally, we investigated the in vivo tumour-promoting effect of MSCs on SKOV3-derived subcutaneous tumours in BALB/c nude mice. We established a subcutaneous tumour model in nude mice. Mice were subcutaneously injected with SKOV3 cells alone or with MSCs/RFP at different ratios. Mice in the WEB2086 group were intraperitoneally injected with the PAFR antagonist WEB2086 at a dose of 1 mg/kg^.^d for the duration of the animal experiments; control mice were injected with DMSO. As shown in Fig. 5a, MSCs alone were not tumourigenic, and compared with the control (SKOV3 cells injected alone), MSCs significantly promoted the growth of SKOV3-derived subcutaneous tumours. The tumour-promoting effect was blocked by the PAFR antagonist WEB2086. However, compared with the SKOV3 + WEB2086 group, the SKOV3 + MSC + WEB2086 group showed a significantly larger tumour volume, suggesting that the PAFR inhibitor could not completely inhibit the effect of MSCs. These data indicated that MSCs can promote the proliferation of OC through the PAF/PAFR pathway, but other mechanisms may also be involved. We also detected PAFR expression in tumours by immunofluorescence. As shown in Fig. 5b, PAFR was highly expressed in tumour tissue. In addition, we prepared frozen tumour sections and performed HE staining to observe the relationship between the tumour parenchyma and stroma. As shown in Fig. 5c, tumour cells were arranged irregularly and tightly when MSCs and SKOV3 cells were co-injected. When SKOV3 cells were injected alone, the tumour cells were less tightly arranged, the stromal area decreased, and larger intracellular spaces were observed in the tissue. Frozen sections were stained with 4′,6-diamidino-2-phenylindole (DAPI) and observed under a confocal microscope. MSCs/RFP could be detected in the tumour stroma in the co-injection groups. These results indicated that when co-injected with SKOV3 cells, MSCs can settle at the injection site and participate in the formation of the tumour stroma. Moreover, there could be cross-talk between MSCs and SKOV3 cells. To determine whether MSCs secreted PAF in vivo, after 4 weeks of treatment, we collected blood from the tail vein and tumour site from mice in each group, and PAF concentrations were measured using a human PAF ELISA kit. As shown in Fig. 5d, the PAF concentration was significantly higher in the tumour site than in peripheral blood; a 10-fold upregulation of PAF was observed when MSCs and SKOV3 cells were co-injected at a ratio of 2:1 compared with that when SKOV3 cells were injected alone. In addition, the PAFR antagonist WEB2086 increased the weight of treated mice, as shown in Fig. 5e, indicating that the inhibitor ameliorated the systemic effects of PAF/PAFR signalling.

## Discussion

Many studies have focused on genetic abnormalities that initiate and drive cancer, and findings have increasingly shown that cancer cells are influenced by their microenvironment to different degrees [17]. The tumour microenvironment comprises stromal cells (such as fibroblasts, MSCs, macrophages, regulatory T cells, endothelial cells and platelets), extracellular matrix components (including inflammatory cytokines, chemokines, matrix metalloproteinases, and other molecules) and exosomes [18]. Cross-talk between tumour cells and the stroma can modulate many tumour processes, including progression, metastasis, and therapeutic resistance [19]. In 1863, Rudolf Virchow first described the possible relationship between inflammation and cancer [20, 21]. Chronic inflammation in the tumour environment is a key factor in the pathogenesis of various malignancies [22]. Inflammation also plays an important role in tumourigenesis, tumour progression, and metastasis.

Among inflammatory factors, PAF is an important pro-inflammatory activator, and PAF/PAFR signalling can activate multiple intracellular signalling pathways. Many studies have reported that PAF can promote the growth of PAFR-positive tumours [11, 23–26]. Our previous studies have shown that PAFR is highly expressed in serous, clear cell and endometrioid OC; PAF can activate PAFR or transactivate epidermal growth factor receptor (EGFR) and initiate multiple downstream signal pathways, specifically through Src/FAK. The PAFR-specific antagonists GB and WEB2086 can block these tumour-promoting effects [12–14].

As an important component of the tumour microenvironment, MSCs were first isolated by Friedenstein [27] et al. in the 1960s. MSCs can be recruited to the tumour site and play an important role in inflammation by secreting various factors and regulating immune function [28]. MSCs also play an important role in tumour progression by differentiating into tumour-associated fibroblasts, inhibiting the immune response, promoting angiogenesis, stimulating epithelial-mesenchymal transition (EMT), promoting tumour metastasis and inhibiting tumour apoptosis [29, 30]. However, the effects of MSCs on the progression of cancer remain controversial [31]. MSCs can play different roles in different tumours or even in the same tumour [32]. Bruno [33] et al. reported that MSCs can induce the necrosis of ovarian tumours and inhibit OCC proliferation by secreting microvesicles. However, Lis [34] et al. reported that co-culture of OCCs and MSCs induces the expression of genes related to cellular adhesion, invasion, migration, proliferation and chemoresistance. Our current study strongly supports the tumour-promoting activity of MSCs in the tumour microenvironment. MSCs promoted the proliferation of OC in vitro and in vivo partly through the PAF/PAFR signalling pathway. Our data showed that MSCs in the tumour microenvironment secreted high levels of PAF and that MSC-CM promoted the proliferation and migration of non-mucinous OCCs (Figs. 1, 2 and 3). In addition, the expression of p-FAK and cyclin D1 in SKOV3 cells was significantly upregulated after treatment with PAF and MSC-CM (Fig. 4). This result is consistent with our previous study [12], which indicated that PAF/PAFR signalling through FAK, PI3K and MMP2 activates multiple downstream signal pathways involved in OCC proliferation and invasion. In vivo, when we co-injected MSCs and OCCs into nude mice, the tumour volume was significantly larger than that when mice were injected with OCCs only, and the PAF concentration of serum from the tumour site serum was much higher in mice co-injected with MSCs and OCCs than in mice injected with OCCs only (5-fold, *p* < 0.05). Moreover, GB and WEB2086 blocked the tumour-promoting effect of MSCs on SKOV3 cells both in vitro and in vivo. However, compared with the SKOV3 + WEB2086 group, the SKOV3 + MSC + WEB2086 group exhibited a significantly larger tumour volume (Fig. 5a), which indicated that the PAFR inhibitor could not completely block the tumour-promoting effect of MSCs. Thus, we speculate that other mechanisms may be involved. Combined with our previous results [12–14], our data indicate that MSCs in the tumour microenvironment can promote the proliferation of non-mucinous OC in vitro and in vivo through the PAF/PAFR signalling pathway. However, we do not know how OCCs induce MSCs to secrete such high concentrations of PAF. Thus, further studies are needed to investigate the mechanism behind the cross-talk between OCCs and MSCs.

## Conclusions

The results of this study shed light on the tumour-promoting effects of MSCs on OC from a molecular perspective. Both in vitro and in vivo analyses suggest that the PAF/PAFR pathway plays an important role in the cross-talk between human MSCs in the inflammatory microenvironment and OC. This finding suggests that MSCs in the tumour microenvironment may be a potential therapeutic target in OC. The specific PAFR inhibitors WEB2086 and GB could be a promising treatment strategy for non-mucinous OC. Thus, a thorough understanding of the role of MSCs in OC is essential. Further studies are required to confirm the molecular mechanism underlying the cross-talk between MSCs and OCCs.
